# Influence of Propolis-Containing Nonwoven PLGA Dressings on Dermatan and Chondroitin Sulfate Dynamics During Burn-Wound Healing

**DOI:** 10.3390/ph19030383

**Published:** 2026-02-27

**Authors:** Kinga Orlińska, Mateusz Stojko, Jakub Włodarczyk, Janusz Kasperczyk, Oskan Tasinov, Diana Ivanova, Mladena Nikolaeva Radeva, Paweł Janik, Katarzyna Komosińska-Vassev, Krystyna Olczyk, Jerzy Stojko, Paweł Olczyk

**Affiliations:** 1 Department of Community Pharmacy, Faculty of Pharmaceutical Sciences in Sosnowiec, Medical University of Silesia in Katowice, 8b Jedności, 41-205 Sosnowiec, Poland; 2 Centre of Polymer and Carbon Materials, Polish Academy of Sciences, 34 M. Curie-Skłodowskiej, 41-819 Zabrze, Poland; mstojko@cmpw-pan.pl (M.S.); jwlodarczyk@cmpw-pan.pl (J.W.); or janusz.kasperczyk@sum.edu.pl (J.K.); 3 Department of Biopharmacy, Faculty of Pharmaceutical Sciences in Sosnowiec, Medical University of Silesia in Katowice, 8 Jedności, 41-200 Sosnowiec, Poland; 4 Department of Biochemistry, Molecular Medicine and Nutrigenomics, Medical University of Varna, 55 Marin Drinov Street, 9002 Varna, Bulgaria; oskan.tasinov@gmail.com (O.T.); divanova@mu-varna.bg (D.I.); 5 Department of Ophthalmology and Visual Sciences, Medical University of Varna, Specialized Eye Hospital—Varna, 55 Marin Drinov Street, 9002 Varna, Bulgaria; mladena.radeva@mu-varna.bg; 6 Institute of Biomedical Engineering, Faculty of Science and Technology, University of Silesia in Katowice, 41-205 Sosnowiec, Poland; pawel.janik@us.edu.pl; 7 Department of Clinical Chemistry and Laboratory Diagnostics, Faculty of Pharmaceutical Sciences in Sosnowiec, Medical University of Silesia in Katowice, 8 Jedności, 41-200 Sosnowiec, Poland; kvassev@sum.edu.pl (K.K.-V.); olczyk@sum.edu.pl (K.O.); 8 Department of Toxicology, Toxicological Analysis and Bioanalysis, Faculty of Pharmaceutical Sciences in Sosnowiec, Medical University of Silesia in Katowice, 30 Ostrogórska, 41-200 Sosnowiec, Poland; jstojko@sum.edu.pl; 9 Faculty of Medical Sciences and Health Sciences, Radom University, Chrobrego 27, 26-600 Radom, Poland; p.olczyk@urad.edu.pl

**Keywords:** burns, chondroitin sulfates, dermatan sulfates, glycosaminoglycans, nonwoven PLGA, propolis, wound healing

## Abstract

**Background/Objectives:** Burn wounds are complex injuries associated with extensive inflammation, extracellular matrix (ECM) damage, and a high risk of impaired tissue remodeling and scarring. Modern wound dressings are expected not only to protect the wound bed but also to actively support the healing process. Biodegradable polymer-based nonwoven dressings incorporating natural bioactive compounds, such as propolis, may favorably influence wound repair. The aim of this study was to evaluate the effect of propolis-containing biodegradable, nonwoven poly(lactide-co-glycolide) (PLGA) dressings on the dynamics of dermatan sulfate and chondroitin sulfate content during burn-wound healing. **Methods:** The present study investigated temporal alterations in sulfated glycosaminoglycans (GAGs), including dermatan and chondroitin sulfates, during the healing of experimentally induced burn wounds in white domestic pigs treated with biodegradable, nonwoven poly(lactide-co-glycolide) (PLGA) dressings containing 5 wt% or 10 wt% of propolis. Control tissue samples were obtained from wounds treated with physiological saline or nonwoven PLGA dressings without propolis. Quantitative analysis of GAG content was performed on days 0, 3, 5, 10, 15, and 21 of the healing process using enzyme-linked immunosorbent assay (ELISA). Statistical differences between groups were assessed by one-way multivariate analysis of variance (MANOVA) followed by Tukey’s post hoc test. **Results:** Propolis-containing biodegradable nonwoven PLGA dressings significantly increased dermatan sulfate and chondroitin sulfate content in the burn wound bed compared to control treatments. The effect was observed at multiple time points and was more pronounced for dressings containing 10 wt% of propolis than for those containing 5 wt%. **Conclusions:** Biodegradable nonwoven PLGA dressings incorporating propolis modulate glycosaminoglycan dynamics during burn-wound healing, indicating enhanced extracellular matrix remodeling and supporting their potential use as bioactive burn wound dressings.

## 1. Introduction

Burn injury is characterized by inflammatory and necrotic changes affecting the skin and adjacent tissues that arise when external energy exceeds the natural protective capacity of the body [1,2,3]. Such injuries typically occur abruptly and constitute a frequent form of trauma that may be associated with systemic complications, including burn disease, and in severe cases may pose a threat to life [4].

Accurate burn classification is essential for selecting adequate first-aid measures and for guiding further therapeutic management of the affected patient [3,5].

In cases of extensive burn injuries, appropriate local therapy is of comparable importance to the management of burn shock, as it significantly influences the subsequent clinical outcome [6].

Currently used pharmaceutical regenerative products physically cover the damaged tissue, meeting the criteria for optimal dressing, but to a moderate degree. The formulations in question do not provide favorable biochemical conditions for the repair process of the damaged tissue matrix, so their effect on the healing phenomenon is limited. Therefore, effective management of cutaneous wounds requires not only external protection of the wound bed but also the application of regenerative preparations containing pharmacologically active, antimicrobial agents.

Key biophysical characteristics of an optimal regenerative dressing—such as biocompatibility, biodegradability, and the ability to regulate wound hydration—may be achieved through the incorporation of biologically active natural materials. Propolis represents one such natural material applied in wound therapy; it is a resin-like substance collected by honeybees from a variety of plant sources and is commonly referred to as bee glue [7,8,9,10].

Propolis exhibits multidirectional biological activity, including anti-inflammatory, antioxidant, antimicrobial, regenerative, and reparative effects on post-burn wounds. The role of propolis in tissue remodeling involves complex biochemical pathways, including stimulation of extracellular matrix (ECM) component transformation, such as glycosaminoglycans, collagen, fibronectin, laminin, and vitronectin, which are essential for tissue repair and restructuring. Its active constituents, particularly flavonoids and phenolic compounds, suppress pro-inflammatory cytokines and enzymes, such as cyclooxygenase and lipoxygenase, thereby reducing inflammation and facilitating tissue remodeling. Propolis has also been shown to modulate matrix metalloproteinase (MMP) activity, maintaining equilibrium between ECM degradation and synthesis.

In addition, its antioxidative capacity protects tissues against oxidative damage, while selected constituents stimulate proliferation and migration of keratinocytes and endothelial cells, supporting re-epithelialization and neovascularization [11,12,13,14,15,16,17,18,19].

Previous studies indicate that propolis enhances burn wound repair by stimulating ECM remodeling, regulating collagen types I and III expression and degradation, and promoting glycosaminoglycan accumulation crucial for granulation and tissue regeneration [20,21].

Propolis has additionally been proposed to stimulate the synthesis of collagen fibers—a process essential for maintaining skin strength, elasticity, and architecture. During wound healing, fibroblasts initially produce type III collagen, which is gradually replaced by type I collagen, providing tensile strength and mechanical stability to newly formed tissue [21,22].

The repair process functions as an interactive sequence of phenomena aimed at restoring both the structural and functional integrity of injured tissue, such as thermally damaged tissue.

Disturbances in the wound-healing process may lead to the development of pathological conditions, including hypertrophic scarring and keloid formation [23].

Wound repair is an interactive sequence of overlapping stages, including hemostasis, inflammation, biosynthesis, and maturation, aimed at restoring structural and functional tissue integrity [24,25].

ECM molecules play key roles throughout wound healing by regulating cell adhesion, migration, signal transduction, and storage of paracrine regulatory factors [25,26,27].

Glycosaminoglycans, collagens, and proteoglycans (PGs), along with paracrine regulatory molecules present within the microenvironment of the injured tissue matrix, actively influence the behavior of stem cells, macrophages, fibroblasts, keratinocytes, and other epidermal cell populations [27,28,29].

Among these, glycosaminoglycans—particularly chondroitin sulfates (CS) and dermatan sulfates (DS)—are crucial regulators of connective tissue integrity, inflammation, oxidative balance, and tissue repair [30,31,32,33,34,35,36,37,38,39,40,41,42]. Poly(lactic-co-glycolic acid) (PLGA) is a biodegradable and biocompatible aliphatic polyester copolymer widely used in pharmaceutical and biomedical applications. PLGA exhibits controlled degradation kinetics that can be precisely modulated by adjusting the ratio of lactic acid to glycolic acid, enabling control of drug release profiles. Extensive scientific research confirms that PLGA is an FDA-approved material for drug delivery systems, characterized by excellent biocompatibility. Due to its structure and physicochemical properties, PLGA serves as an ideal platform for manufacturing advanced wound-dressing materials that combine the biological functionality of a drug carrier with the structural properties of a dressing material, enabling sustained and controlled release of pharmaceutical compounds at the wound site [43,44,45]. Electrospinning is a nanofabrication technique enabling the production of nanofibers with highly desirable structural and functional properties. The process involves applying an electric field to a polymer solution or molten polymer, creating a charged liquid jet transported to a collector of opposite polarity, where fibers are deposited. In solution electrospinning, the polymer (or mixture of polymer with active compound) is dissolved in an appropriate organic solvent (or solvent mixture), forming a solution subjected to the electrospinning process. For wound dressing applications, this technique enables the formation of nonwoven dressing materials characterized by high porosity, large surface area, microporous structure, and the ability to control the release of active compounds. Electrospinning enables precise control over morphological and chemical parameters of the resulting nanofiber structures [46,47,48]. Although the biological activity of propolis and the properties of PLGA-based dressings are well documented, their combined influence on the dynamics of dermal glycosaminoglycans during burn-wound healing remains insufficiently characterized. Therefore, the primary objective of this study was to evaluate temporal changes in selected glycosaminoglycan types, including chondroitin sulfates and dermatan sulfates, within the beds of experimentally induced burn wounds in white domestic pigs treated with nonwoven PLGA dressings containing 5% or 10% propolis.

## 2. Results

The levels of individual glycosaminoglycans in normal skin and in the wound bed of burn injuries treated on successive days with nonwoven PLGA-enriched propolis or physiological saline were determined based on glycosaminoglycan concentrations measured in tissue homogenates. The results presenting the content of the tested glycosaminoglycan fractions in individual tissue samples are presented in Table 1 and Table 2.

### 2.1. Dermatan Sulfates

**Figure 1:**
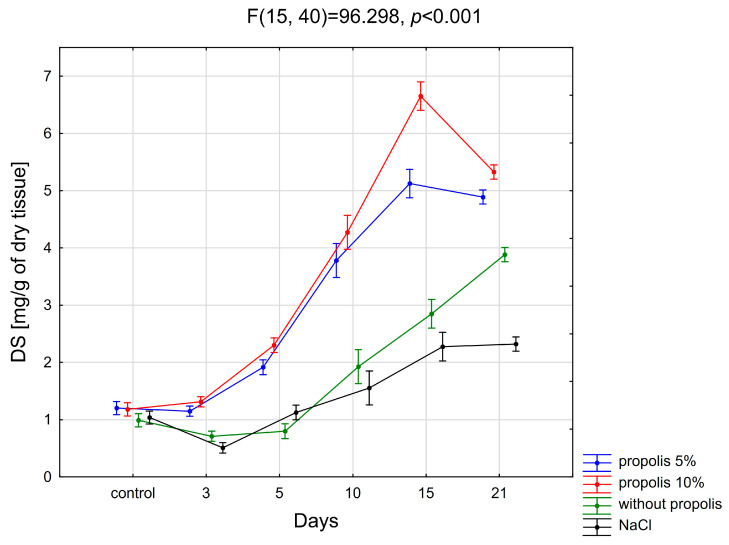
Changes in dermatan sulfate (DS) content in burn wound tissue during healing in animals treated with nonwoven PLGA dressings containing propolis (5% and 10%), nonwoven PLGA without propolis, or physiological saline (NaCl). Values are presented as mean ± SD (N = 3). Time points represent predefined sampling days (0, 3, 5, 10, 15, and 21) and are not equally spaced in time. Statistical significance was determined using one-way multivariate analysis of variance (MANOVA) followed by Tukey’s post hoc test (*p* < 0.05).

Figure 1 illustrates temporal changes in dermatan sulfate content observed during the healing of experimentally induced burn wounds treated on consecutive days with nonwoven PLGA dressings containing 5 wt%, 10 wt%, or no propolis, as well as wounds rinsed with physiological saline solution.

#### 2.1.1. Propolis 5%

The statistical analysis of the obtained results reveals a significant increase in the content of dermatan sulfates within the damaged tissue during the healing process of post-burn wounds treated with a nonwoven PLGA dressing containing 5% propolis. Specifically, on days 5, 10, 15, and 21 post-injury, DS levels were markedly elevated compared to those found in intact skin (*p* < 0.001).

It was also found that the content of DS in the healing-wound bed treated with the discussed biodegradable dressing differed significantly between days 3 and 5, 5 and 10, and 10 and 15 (*p* < 0.001). However, no differences were found in the content of dermatan sulfates between normal tissue (day 0) and damaged tissue on day 3 after the wound infliction, and—in the area of burnt skin—between days 15 and 21 of the wound-healing process.

#### 2.1.2. Propolis 10%

The statistical analysis of the obtained results reveals a notable increase in the content of dermatan sulfates within post-burn wounds undergoing treatment with a nonwoven PLGA dressing containing 10% of propolis. Specifically, on the 5th, 10th, 15th, and 21st days of treatment, DS levels are significantly higher in the post-burn wounds compared to those observed in the control skin (*p* < 0.001).

Moreover, it was found that the content of DS in the wound bed differed significantly between days 3 and 5, 5 and 10, 10 and 15, and 15 and 21 of post-burn-wound healing (*p* < 0.001).

#### 2.1.3. Without Propolis

As can be seen from Figure 1, utilizing nonwoven PLGA dressing without propolis results in a noteworthy augmentation of DS content within the injured tissue. Specifically, on the 10th, 15th, and 21st days of the experiment, DS levels exhibit a significant increase compared to those found in healthy skin (*p* < 0.001).

The statistical evaluation of the obtained results showed differences in the content of dermatan sulfates in the burn wound treated with a propolis-free nonwoven PLGA dressing on individual days of treatment. It was shown that the content of DS in the wound differed significantly on days 5 and 10, 10 and 15, and 15 and 21 (*p* < 0.001).

#### 2.1.4. Physiological Salt Solution

Statistical analysis revealed that the content of dermatan sulfates in the post-burn wound treated with physiological salt solution was significantly lower compared to the accumulation of DS in normal skin on the 3rd days (*p* < 0.01) and significantly higher on the 10th (*p* < 0.01), 15th (*p* < 0.001) and 21st days (*p* < 0.001) after the exposure to the thermal factor. However, there was no statistically significant difference in the DS content between the post-burn wound on the 5th day of its healing and healthy skin.

The conducted research showed that the content of DS in a burn wound washed with physiological saline (NaCl) solution differed significantly between days 3 and 5 (*p* < 0.001), 5 and 10 (*p* < 0.05), and 10 and 15 (*p* < 0.001).

#### 2.1.5. Comparison of Different Treatment Methods

As can be seen from the present study, post-burn wound treatment with a biodegradable nonwoven PLGA dressing containing 5% propolis leads to a consistent elevation in DS levels within the wound bed. Specifically, on the 3rd, 5th, 10th, 15th, and 21st days of the repair process, DS content demonstrates a significant increase compared to the values observed in wounds treated with a physiological salt solution on the same day (*p* < 0.001). Statistical analysis of the results obtained in this study also showed that the content of dermatan sulfates during the healing of wounds treated with a nonwoven PLGA dressing containing 10% propolis was significantly higher in post-burn wounds on all days on which skin samples were taken, i.e., days 3, 5, 10, 15 and 21, compared to the values observed in places of tissue damage on the same days of therapy in wounds that were treated with NaCl solution (*p* < 0.001). There was no significant effect of treatment with a nonwoven PLGA dressing without propolis compared to the treatment with NaCl solution, and on the content of DS in post-burn wounds on the 3rd, 5^th^, and 10th days of the experiment, while on the 15th (*p* < 0.01) and 21st (*p* < 0.001) days a significant increase in the content of the glycans in question, compared to the content of DS in the burn wound treated with physiologic salt solution, was observed.

### 2.2. Chondroitin Sulfates

**Figure 2:**
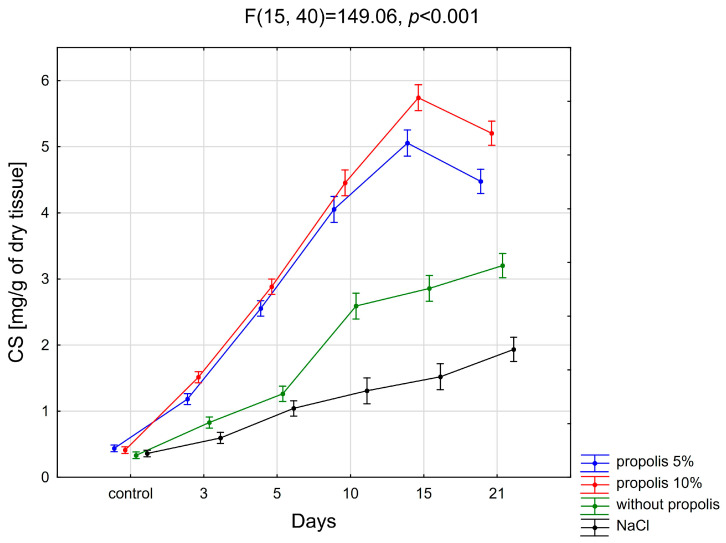
Changes in chondroitin sulfate (CS) content in burn wound tissue during healing in animals treated with nonwoven PLGA dressings containing propolis (5% and 10%), nonwoven PLGA without propolis, or physiological saline (NaCl). Values are presented as mean ± SD (N = 3). Time points represent predefined sampling days (0, 3, 5, 10, 15, and 21) and are not equally spaced in time. Statistical significance was determined using one-way multivariate analysis of variance (MANOVA) followed by Tukey’s post hoc test (*p* < 0.05).

The dynamics of changes in the content of chondroitin sulfates during the healing of experimental burn wounds in subsequent days of treatment with nonwoven PLGA dressings containing 5%, 10% and without propolis, as well as during subsequent days of washing them with physiological salt solution, are shown in Figure 2.

#### 2.2.1. Propolis 5%

As a result of the conducted research, it was shown that on all days (3, 5, 10, 15, 21) of the experiment, the content of chondroitin sulfates in the burn wound treated with a nonwoven PLGA dressing containing 5% of propolis was significantly higher compared to the content of these compounds in normal skin (*p* < 0.001). Statistical evaluation of the results regarding the content of chondroitin sulfates in the bed wound treated with a biodegradable dressing enriched with 5% propolis showed significant statistical differences between the 3rd and 5th, 5th and 10th, 10th and 15th, and 15th and 21st days of wound healing (*p* < 0.001).

#### 2.2.2. Propolis 10%

The statistical evaluation showed that on all subsequent days (3, 5, 10, 15, 21) of the experiment, there was a significant increase in the content of chondroitin sulfates in the wound treated with a biodegradable dressing enriched with 10% propolis, compared to the amount of these glycans occurring in healthy skin (*p* < 0.001).

It was found that the content of chondroitin sulfates differed significantly between the 3rd and 5th, 5th and 10th, 10th and 15th, and 15th and 21st days of dressing the wound with a nonwoven PLGA dressing containing 10% propolis (*p* < 0.001).

#### 2.2.3. Without Propolis

Statistical analysis of the obtained results showed that in the case of a wound treated with a nonwoven PLGA dressing without propolis, there was a statistically significant difference in the content of chondroitin sulfates on the 3rd, 5th, 10th, 15th, and 21st day of the experiment, compared to the amount of CS characterizing healthy skin (*p* < 0.001).

A statistically significant difference was found between the 3rd and 5th (*p* < 0.01), 5th and 10th (*p* < 0.001), and 15th and 21st (*p* < 0.05) days of post-burn wound treatment with a biodegradable dressing without propolis.

#### 2.2.4. Physiological Salt Solution

Based on the results obtained, it was shown that on the 5th, 10th, 15th, and 21st days of healing of the burn wound washed with physiological salt solution, there is a significant increase in the content of chondroitin sulfates compared to the content of these glycans in the skin constituting the control material (*p* < 0.001).

In the case of washing the burn wound with NaCl solution, it was observed that the CS content differed significantly only between the 3rd and 5th and 15th and 21st days of wound healing (*p* < 0.01).

#### 2.2.5. Comparison of Different Treatment Methods

The statistical assessment of the impact of the applied method of a burn wound treating on the content of chondroitin sulfates showed that it was significantly higher in wounds treated with a biodegradable dressing containing 5% and 10% propolis (*p* < 0.001)—on all days of the repair process, and—in wounds treated polymer nonwoven without a dressing (*p* < 0.001)—on days 10, 15 and 21 of the experiment, compared to the content of these compounds in the wound treated with NaCl on all days of the experiment.

## 3. Discussion

Burn wounds were deliberately selected as the experimental model due to their complex and highly dynamic healing process, which involves an intense inflammatory response, extensive extracellular matrix disruption, and a high risk of abnormal tissue remodeling and scar formation [25,26,27]. Unlike many other acute wounds, thermal injuries are characterized by prolonged inflammation, oxidative stress, and profound alterations in the composition and organization of glycosaminoglycans and proteoglycans within the wound bed [29,30,31]. These features make burn wounds particularly sensitive to biologically active dressings and provide a relevant model for evaluating interventions aimed at modulating extracellular matrix remodeling [25,26,27,31].

The enhancement of chondroitin sulfate and dermatan sulfate production observed in propolis-treated wounds may be associated with the anti-inflammatory properties attributed to propolis in previous studies. Propolis is rich in bioactive compounds, such as flavonoids, phenolic acids, and terpenes, which significantly contribute to its anti-inflammatory effects. These compounds have been reported to modulate the production of pro-inflammatory cytokines, such as tumor necrosis factor alpha (TNF-α), interleukin 1 beta (IL-1β), and interleukin 6 (IL-6), which are the key players in the inflammatory process. Additionally, propolis has been described as exhibiting antioxidant properties that may contribute to the reduction in oxidative stress associated with inflammation [49,50,51].

One of the mechanisms proposed in the literature for the anti-inflammatory activity of propolis involves modulation of the nuclear factor kappa B (NF-κB) pathway, a crucial regulator of inflammation and immune responses. Propolis has also been reported to influence the mitogen-activated protein kinase (MAPK) pathway, further contributing to its anti-inflammatory effects. These pathways are known to regulate inflammatory responses and tissue homeostasis, processes that are important for wound healing and extracellular matrix remodeling [50,51].

Propolis has been shown to provide various health benefits, including reducing inflammation associated with cancer, managing oral inflammation and infections such as periodontitis, and alleviating inflammation related to metabolic syndrome, including obesity and diabetes. It also protects against inflammation-induced organ toxicity and modulates immune responses, balancing pro- and anti-inflammatory effects, which are crucial for tissue integrity and repair [49,50,51].

The increased production of CS and DS observed in propolis-treated burn wounds may reflect biological effects consistent with the anti-inflammatory properties attributed to propolis. It is possible that the biological activity of propolis contributes to creating conditions favorable for tissue regeneration. This comprehensive anti-inflammatory activity supports the therapeutic use of propolis in enhancing wound healing and managing other inflammatory conditions [49,52,53].

The profile of qualitative and quantitative changes in glycosaminoglycans has already been described, shaped by the mechanisms of subsequent phases of healing of burn wounds supplied with propolis [13,14]. It should be emphasized that although increased levels of chondroitin sulfates and dermatan sulfates may also be observed in scar tissue, their evaluation in the present study was performed in a dynamic manner across consecutive stages of wound healing. The temporal pattern of CS and DS accumulation followed by a subsequent decrease reflects extracellular matrix remodeling rather than scar formation alone.

However, it should be emphasized that biodegradable electrospun nonwoven dressings releasing propolis have already been investigated and discussed in previous studies, including those reported by Stojko et al. [44] and Stojko et al. [47]. The novelty of the present work lies in a biochemical evaluation of the wound-healing response, focused on the dynamics of dermatan sulfates and chondroitin sulfates accumulation in the burn wound bed at consecutive stages of the repair process. In contrast to prior reports primarily addressing the material concept and general wound-healing potential, our study provides quantitative insight into extracellular matrix remodeling by monitoring sulfated glycosaminoglycans as molecular indicators of tissue repair. Therefore, the current results complement and extend the existing evidence on propolis-loaded electrospun dressings by adding a detailed analysis of GAG-related mechanisms involved in burn wound regeneration.

What is particularly important is that both the natural product itself with known medicinal properties, i.e., propolis, and the electrospinning product with numerous applications in medicine, can favorably promote tissue repair processes. The conducted research showed that during burn-wound healing, the content of dermatan sulfates in the burn wound bed changes, depending on the method of skin injury dressing. In tissue samples obtained from the site of thermal damage, during subsequent days of treatment with nonwoven PLGA dressings containing 5% or 10% propolis, the DS content increased significantly in relation to the content of the discussed glycans in the control sample, i.e., tissue not subjected to burns, and then—starting from the 15th day of the experiment—the content of the glycan in question began to drop.

These changes occurred with varying intensity, depending on the concentration of propolis (5% or 10%) in the dressing, and were more intense in the case of a higher concentration of the mentioned active substance. The nonwoven polymer, not containing propolis, stimulated a slight downward trend in the DS content until day 5 of the experiment, after which there was a statistically significant increase in the content of the glycans in question, starting from the third day of the experiment. The obtained results allowed us to demonstrate the advantage of dressings containing propolis over dressings without propolis in affecting dermatan sulfates involved in the repair process of damaged tissues.

The observed pattern of changes in dermatan sulfate content within the matrix of thermally injured tissue treated with nonwoven PLGA dressings with propolis was comparable to the course of natural wound healing observed in wounds treated with physiological saline, without pharmacological intervention. The discussed glycan fraction dominates in normal skin, constituting 78% of all sulfated GAGs, during the healing process—the DS content, both in the wound fluid and in the tissue, increases significantly. This increase reflects the physiological involvement of dermatan sulfates in provisional matrix formation and tissue remodeling during normal wound healing rather than scar maturation. It should be noted that just skin, being the main place of DS occurrence in the body, gave rise to the name of the discussed glycans, as derived from the word dermis [38,39,40,41,42,54,55,56,57,58,59,60,61,62].

The present study also showed that neither the propolis-free nonwoven PLGA nor the physiological salt had any significant influence on DS transformations during the healing process, which was manifested by the low intensity of DS content increment in the damaged tissue, simultaneously reflecting the slow course of the tissue repair process.

Similar results to those obtained in the present study—an increase in the content of dermatan sulfates and chondroitin sulfates in the wound bed—were also reported by Siméon et al. [61]. In their experiment, they exposed wounds to a complex: tripeptide (gly-his-lys)—Cu^2+^—which stimulates the tissue repair process by exhibiting growth factor-like properties in relation to differentiating cells, chemotactic activity towards monocytes, macrophages, or mast cells, enhancement of angiogenesis, synthesis of ECM components, and acceleration of reepithelialization.

The results obtained in this study also correspond to the results of research by other authors regarding the effect of an apitherapeutic agent—propolis—on post-burn wounds [14]. In the mentioned studies, a systematic increase in the dermatan sulfate content was observed from the day of tissue damage, and then—from about day 15—a decrease in the DS content.

The glycan in question, whose expression increases during the damaged tissue repair, is responsible for the stimulation of leukocyte adhesion to endothelial cells via intercellular adhesion molecule 1 (ICAM-1) [14,61,62]. Moreover, DS, but also HS/H, interacting with growth factors, such as insulin-like growth factor (IGF), fibroblast growth factor (FGF), and hepatocyte growth factor/scatter factor (HGF/SC), actively modulate the development, viability, motion, and differentiation of cells (i.e., epithelial and endothelial), being phenomena particularly needed in the course of wound regeneration [63].

DS also interacts with other growth factors, i.e., FGF-2 and FGF-7 [14,58]. The binding of DS to FGF-2 promotes the recruitment of inflammatory cells stimulates fibroblast proliferation as well as endothelial cell proliferation and migration, enabling the angiogenesis process. The mentioned growth factor also increases the expression of type I and III collagen. On the other hand, FGF-7 stimulates the regeneration of keratinocytes [64,65].

Moreover, the phenomenon of increased decorin (small leucine-rich proteoglycan containing glycosaminoglycan chain—consisting of either CS or DS) expression during the healing process has been described, which, considering the physiological role of this PG, promotes, among others, the formation of collagen fibers with desired biomechanical properties at the site of tissue repair [66].

Another class of glycosaminoglycans involved in the wound-healing process comprises chondroitin sulfates.

This study demonstrated that the content of the discussed GAGs, isolated from burn wounds, increases during the healing process, regardless of the method of dressing the wound. The greatest intensity of the increase in the content of chondroitin sulfates, progressing until the 15th day of the healing process, followed by a sudden decrease in the content of this GAG fraction, was demonstrated in the case of wound beds treated with a dressing enriched with propolis. The severity of the changes was greater when 10% propolis was used. In both of the above-mentioned cases (5% and 10% propolis), the CS content of burn wounds differed statistically significantly from the changes observed in the case of wounds treated with NaCl. Changes in the CS content in the burnt tissues treated with propolis-free nonwoven PLGA showed a similar tendency to those treated with NaCl.

The results of this work correspond to the research results of other authors. Thus, Siméon’s research [61] demonstrated a systematic increase in the content of chondroitin sulfates, similar to dermatan sulfates, beginning immediately after tissue injury and progressing during the granulation phase of healing.

As in the case of the studies by Siméon [61], in the presently described impact of nonwoven PLGA containing 5% or 10% propolis on the transformation of chondroitin sulfates during the wound-healing process, an analogy can be indicated regarding previous research by Olczyk et al. [14], assessing the effect of propolis on CS transformations in the burn wound bed during the tissue healing process. The latter showed an increase in the content of chondroitin sulfates in the burn wound bed, progressing from the moment of injury until about the 15th day after its occurrence, and then a decrease in the content of the discussed glycan [14].

Both chondroitin sulfates and dermatan sulfates interact with growth factors, cytokines, and extracellular matrix macromolecules and thereby participate in cellular processes such as proliferation, migration, and adhesion. The extent of interaction between these glycans and growth factors or other signaling molecules depends on both the number and spatial distribution of sulfate groups within the CS and DS chains.

CS/DS furthermore regulates the expression of the nitrogen monoxide by endothelial cells needed for the stimulation and regulation of the new capillaries’ formation in the angiogenesis process [31,60,67].

Chondroitin sulfate and dermatan sulfate proteoglycans also participate in collagen polymerization, while DS also plays the pivotal role in fibrogenesis [53,66].

It should also be noted that poly(lactide-co-glycolide) (PLGA) is a biodegradable polymer, and its gradual degradation in the wound environment may contribute to beneficial modulation of the local wound microenvironment. The degradation products of PLGA, including lactic and glycolic acids, may influence local pH and hydration, thereby supporting physiological healing mechanisms. In addition, the biodegradable nature of PLGA-based dressings may reduce the need for frequent dressing changes, limiting mechanical disturbance of the wound bed and decreasing the risk of damage to newly formed tissue.

The increased synthesis of individual GAGs in the burn wound bed, as demonstrated in the case of the use of nonwoven PLGA dressings incorporated with propolis, indicates the intensification of the healing processes stimulated by the above-mentioned medicinal substance.

It should be emphasized that thermal damage is difficult to heal and may also result in the formation of a scar. Therefore, the microenvironment of the healing wound bed, arranged during the therapeutic procedure, is so important, both in terms of the process itself and its final effect [68].

Despite these promising findings, several limitations should be acknowledged, and important considerations regarding the safety and standardization of propolis-based materials should be addressed. It should be emphasized that specific intracellular signaling pathways were not directly evaluated in the present study; therefore, the proposed mechanisms are based on previously published data concerning propolis and its bioactive constituents.

### 3.1. Limitations of the Study

This study has several limitations that should be considered when interpreting the results. First, the evaluation was focused on biochemical markers of extracellular matrix remodeling (dermatan sulfates and chondroitin sulfates), while clinical endpoints such as wound closure rate, re-epithelialization, and scar formation were not quantitatively assessed. Second, the sample size in each group was limited (N = 3), which may affect the statistical power of the analysis. Third, although the formulation parameters of the electrospun dressings were provided, a broader physicochemical characterization of the nonwoven PLGA structure (e.g., morphology and mechanical properties) was beyond the scope of this work. Future studies integrating biochemical, histological, and macroscopic wound-healing assessments are warranted.

### 3.2. Safety and Standardization of Propolis-Based Materials

Propolis is a natural product with well-documented biological activity; however, its chemical composition may vary depending on geographic and botanical origin, extraction method, and storage conditions. This variability highlights the importance of standardization and quality control for propolis-based biomaterials intended for biomedical applications. In addition, although propolis is generally considered safe, hypersensitivity reactions have been reported in susceptible individuals, which should be taken into account in further translational studies. Therefore, future research should address formulation reproducibility, batch-to-batch consistency, and safety evaluation in order to support potential clinical applicability.

### 3.3. Future Perspectives

Future studies should integrate the biochemical findings presented in this work with histological and macroscopic assessments of wound healing. In particular, quantitative evaluation of wound closure rate, re-epithelialization, and scar formation would strengthen the clinical relevance of propolis-loaded nonwoven PLGA dressings. Moreover, the release kinetics of propolis from the electrospun matrix should be investigated to better correlate local exposure with biological response. Additional in vitro safety testing, including cytotoxicity and irritation potential, would further support translational applicability. Finally, standardization of propolis composition and batch-to-batch reproducibility should be addressed in order to ensure comparability and future clinical implementation.

## 4. Materials and Methods

### 4.1. Biological Material

The study protocol was approved by the Ethics Committee of the Medical University of Silesia in Katowice, Poland (no. LKE111/2014). Animal experiments were conducted at the Center for Experimental Medicine of the Medical University of Silesia in Katowice. Domestic pigs were kept under the same zoohygienic conditions throughout the experiment to ensure full psychophysical comfort and minimize stress. They were fed a complete R 233 mixture. The mixture covered 100% of the animals’ nutritional requirements for energy, protein, amino acids, vitamins, and minerals. No special diet or supplementation that could affect the wound-healing process was used. Post-burn skin wounds were induced according to the Hoekstra standard model [57]. Four 16-week-old female pigs (35–40 kg) were used in the study. After premedication (atropine sulfate, 0.05 mg/kg body weight, s.c.; ketamine hydrochloride, 3 mg/kg body weight, i.v.; xylazine hydrochloride, 1 mg/kg body weight, i.v.), the animals were subjected to general anesthesia with thiopental sodium salt (5 mg/kg body weight, i.v.). After achieving deep analgesia, burn wounds (1.5 cm × 3 cm) were inflicted by applying a Lancetron D electrode heated to 170 °C for 20 s at symmetrical sites (9 wounds on each side of the animal).

Tissue samples for biochemical analyses were collected from the burn wound bed on days 3, 5, 10, 15, and 21 after injury in each experimental group (nonwoven PLGA dressings containing 5 wt% or 10 wt% propolis, propolis-free nonwoven PLGA dressing, or physiological saline treatment). Control samples (day 0) were collected from intact skin prior to burn induction. All experimental groups were assessed at the same predefined time points. All tissue specimens were stored at −75 °C until further analysis.

### 4.2. Materials

Poly(L-lactide-co-glycolide) (PLGA) 85:15 copolymer was used for nonwoven PLGA fabrication. Propolis was obtained from Apipol-Farma (Myślenice, Poland). The preparation was obtained from a single production batch to ensure consistency throughout the experiment. 1,1,1,3,3,3-hexafluoro-2-propanol (HFIP) was purchased from Sigma-Aldrich (Merck KGaA, Darmstadt, Germany). Chloroform and methanol were purchased from Avantor Performance Materials Poland S.A. (Gliwice, Poland). Electrospinning was performed using a TL-Pro-BM unit (Tong Li Tech, Shenzhen, China) and a Harvard Apparatus PHD Ultra 4400 syringe pump (Harvard Apparatus, Cambridge, MA, USA). Dermatan sulfate and chondroitin sulfate levels were quantified using commercial ELISA kits: Porcine Dermatan sulfate (DS) ELISA kit (AMSBIO, Abingdon, Oxfordshire, UK; catalog no. AMS.E07D0036) and Enzyme-linked Immunosorbent Assay Kit for Chondroitin Sulfate (CS) (Cloud-Clone Corp. Katy, TX, USA; catalog no. CEA723Ge).

### 4.3. Production of Nonwoven PLGA

The nonwovens were made in cooperation with the Department of Biopharmacy of the Faculty of Pharmaceutical Sciences in Sosnowiec, the Medical University of Silesia in Katowice, and the Center for Polymer and Polycarbonate Materials in Zabrze, Polish Academy of Sciences.

Poly(L-lactide-co-glycolide) 85:15 was synthesized according to the method described in the literature [69] in bulk via the ring opening polymerization (ROP) of L-lactide and glycolide with the use of zirconium (IV) acetylacetonate (Zr(Acac)4) as a low-toxic initiator (initiator to monomer I/M molar ratio: 1:600). The reaction vessel was conditioned in an oil bath (periodically stirring) at 130 °C for 24 h and then at 115 °C for 72 h. The copolymers thus obtained were purified by dissolution in chloroform (Avantor Performance Materials Poland S.A., Gliwice, Poland) and precipitation into cold methanol (Avantor Performance Materials Poland S.A., Gliwice, Poland) in order to remove the unreacted monomers, followed by drying under a vacuum at room temperature to constant weight.

Samples without active compound were prepared by dissolution of PLGA 85/15 (6% *w*/*w*) in 1,1,1,3,3,3-hexafluoro-2-propanol (HFIP) (Sigma Aldrich, Merck KGaA, Darmstadt, Germany). Samples containing propolis were prepared by dissolution of PLGA 85/15 (6% *w*/*w*) and propolis (Apipol-Farma, Myślenice, Poland) (5% (*w*/*w*) and 10% (*w*/*w*) propolis concentration relative to the polymer) in HFIP. Prepared solutions were used for obtaining nonwovens with the TL-Pro-BM electrospinning unit (Tong Li Tech, Shenzhen, China). The device was equipped with two high-voltage power supplies. Potential difference was 27 kV. Positive electrical potential was applied to the spinneret (2 × 20 G steel needle), and negative potential to the collector (steel mandrel ϕ = 27 mm, rotating at a rate of 800 RPM). Tip-to-collector distance was set to 21 cm. Polymer solutions were dosed to the spinning nozzle through a capillary at 3 mL/h by using a Harvard Apparatus PHD Ultra 4400 (Harvard Apparatus, Cambridge, MA, USA) syringe pump. The average temperature inside the spinning chamber was 17 ± 1 °C, while the relative humidity was about 48%. PLGA nonwovens were obtained in the form of 27 cm × 8.5 cm sheets. The obtained nonwovens were dried under a vacuum at room temperature to constant weight.

### 4.4. Isolation of Glycosaminoglycans

Isolation of glycosaminoglycans from homogenized, dehydrated, and defatted tissue samples was performed using the Scott method [58], modified by Van Amerongen et al. [59]. The isolation of GAGs was carried out at the Department of Clinical Chemistry and Laboratory Diagnostics, Faculty of Pharmaceutical Sciences in Sosnowiec, Medical University of Silesia in Katowice.

### 4.5. Assessment of Glycosaminoglycan Content

To assess the content of dermatan sulfates, all elements of the kit and tissue samples were brought to room temperature (20–25 °C) before use. Since the range of detectable GAG concentrations was unknown, a preliminary detection trial was conducted. The washing solution for the microplate wells was prepared by diluting 10 mL of concentrate in 990 mL of distilled water, stable for 2 weeks at 2–8 °C. Other components were ready to use.

For chondroitin sulfate content assessment, all elements of the kit and tissue samples were brought to room temperature (20–25 °C). Before the assay, the standard was diluted in 2 mL of CS and left at room temperature for 10 min, then gently shaken. The standard concentration in the stock solution was 10,000 pg/mL. Five eppendorf tubes containing 0.6 mL of standard diluent were prepared, and serial dilutions were performed, resulting in concentrations of 10,000 pg/mL, 2500 pg/mL, 625 pg/mL, 156.25 pg/mL, and 39.06 pg/mL. The last tube contained only the diluent as a blank. Reagents A and B were briefly centrifuged and then diluted 100-fold to the working concentration. The washing solution was prepared by diluting 20 mL of concentrate (30×) in 580 mL of distilled water to obtain 600 mL of 1× solution.

A 96-well plate was designed to allocate wells for standard solutions, blanks, and tissue samples. Five wells were designated for each standard dilution, and one well was designated for the blank. 100 µL for DS or 50 µL for CS of each standard dilution, blank, and tissue samples were added to the designated wells. Then, 10 µL of stabilizing solution and 50 µL of conjugate (except the control) for DS, or 50 µL of detecting reagent A for CS, were immediately added to each well. The plate was gently shaken and sealed with foil, then incubated for 1 h at 37 °C. After incubation, the plate was brought to room temperature and mixed. The remaining solution was discarded, wells were washed with the washing solution, left for 1–2 min at room temperature, and excess liquid was removed by blotting. This washing step was repeated three times for CS and five times for DS. 50 µL of substrate A and B for DS, or 100 µL of detecting reagent B for CS, were added to each well and incubated at 37 °C. For CS, 90 µL of substrate solution was added, sealed, and incubated for 10–20 min at 37 °C. Finally, 50 µL of stopping solution was added to all wells, changing the solution color to yellow. The contents were mixed by tapping, and an immediate reading at 450 nm was taken using a microplate reader (Infinite® M200, Tecan Group Ltd., Männedorf, Switzerland).

### 4.6. Identification of Glycosaminoglycans

The content of individual glycosaminoglycans in tissue sample homogenates was determined with the enzyme-linked immunosorbent method (ELISA), using the following tests: Porcine Dermatan sulfate (DS) Elisa kit, catalog number AMS.E07D0036, by Amsbio (Abingdon, Oxfordshire, UK); and Enzyme-linked Immunosorbent Assay Kit for Chondroitin Sulfate (CS), cat. no. CEA723Ge from Cloud-Clone Corp. (Katy, TX, USA).

### 4.7. Statistical Analysis

The compliance of the distributions of individual groups of results with the normal distribution was verified upon implementation of the Shapiro–Wilk W test. Statistical analysis was performed using one-way multivariate analysis of variance (one-way MANOVA) to test differences between control and treated groups at each time point, followed by Tukey’s post hoc test. The assumption of homogeneity of variance (sphericity) was checked using Mauchly’s test. Due to the deviation from sphericity, a multivariate approach was applied, as well as contrast analysis to compare the average results obtained on individual days of the experiment. Differences were considered statistically significant at *p* < 0.05. The arithmetic mean and standard deviation were selected as descriptive statistics. The analysis was performed using Statistica, version 13 (TIBCO Software Inc., Palo Alto, CA, USA, 2017). 

## 5. Conclusions

The pharmacological efficacy of biodegradable nonwoven PLGA dressings containing 5% and 10% propolis in the healing of skin burn wounds was assessed by studying the dynamics of the content changes in dermatan sulfates and chondroitin sulfates isolated from the wound beds. A comparison with the content changes in CS and DS isolated from wounds treated with nonwoven PLGA dressings without propolis revealed the beneficial effects of propolis-infused nonwoven PLGA fabric on tissue wound healing.

The enhanced healing process of burn wounds facilitated by nonwoven PLGA dressings infused with natural products was evidenced by the intensified and expedited accumulation of glycosaminoglycans within the wound bed.

The present study shows the multifaceted benefits of utilizing natural product-infused dressings.

## Figures and Tables

**Table 1:** The content of dermatan sulfates in homogenates of tissue samples obtained from healthy skin and from burn wound beds on subsequent days of the wound-healing process.

Content of Dermatan Sulfates [mg/g of Dry Tissue] in the Burn Wounds				
Days of Experiment	NaCl (*N* = 3)	Nonwoven PLGA Without Propolis (*N* = 3)	Nonwoven PLGA with 5% Propolis (*N* = 3)	Nonwoven PLGA with 10% Propolis (*N* = 3)
Day 0 (normal skin)	1.037000 ± 0.039345	0.990000 ± 0.031225	1.202000 ± 0.113371	1.181000 ± 0.117987
Day 3	0.506667 ± 0.042028	0.709000 ± 0.012530	1.147667 ± 0.040612	1.313000 ± 0.120801
Day 5	1.126000 ± 0.036290	0.799000 ± 0.007211	1.914333 ± 0.088484	2.299667 ± 0.167046
Day 10	1.553000 ± 0.042332	1.925000 ± 0.127472	3.779667 ± 0.098338	4.271667 ± 0.414126
Day 15	2.274667 ± 0.033531	2.849333 ± 0.094875	5.124667 ± 0.138399	6.652333 ± 0.333163
Day 21	2.320000 ± 0.046605	3.883667 ± 0.102237	4.889667 ± 0.113006	5.325667 ± 0.098450

The presented numerical values represent the arithmetic mean of three measurements made each day ± standard deviation, considering the method of wound dressing.

**Table 2:** The content of chondroitin sulfates in homogenates of samples obtained from healthy skin and burn wounds on subsequent days of the wound-healing process.

The Content of Chondroitin Sulfates [mg/g of Dry Tissue] in a Burn Wound				
Days of Experiment	NaCl (*N* = 3)	Nonwoven PLGA Without Propolis (*N* = 3)	Nonwoven PLGA with 5% Propolis (*N* = 3)	Nonwoven PLGA with 10% Propolis (*N* = 3)
Day 0 (normal skin)	0.359000 ± 0.047843	0.334000 ± 0.020664	0.437000 ± 0.052735	0.410333 ± 0.016503
Day 3	0.596667 ± 0.032254	0.828000 ± 0.073750	1.182333 ± 0.063658	1.513333 ± 0.073214
Day 5	1.042333 ± 0.067159	1.261333 ± 0.092986	2.554000 ± 0.079681	2.882667 ± 0.105268
Day 10	1.307667 ± 0.030925	2.589667 ± 0.084524	4.052000 ± 0.090316	4.454000 ± 0.264704
Day 15	1.521333 ± 0.035529	2.857000 ± 0.084149	5.055000 ± 0.140275	5.742333 ± 0.243192
Day 21	1.934000 ± 0.040361	3.202333 ± 0.053725	4.474667 ± 0.162112	5.204333 ± 0.212079

The presented numerical values represent the arithmetic mean of three measurements made each day ± standard deviation, considering the method of wound dressing.

## Data Availability

The original contributions presented in the study are included in the article; further inquiries can be directed to the corresponding author.
